# Cost recovery of NGO primary health care facilities: a case study in Bangladesh

**DOI:** 10.1186/1478-7547-8-12

**Published:** 2010-06-09

**Authors:** Khurshid Alam, Shakil Ahmed

**Affiliations:** 1 James P. Grant School of Public Health, BRAC University, Dhaka 1212, Bangladesh; 2 Health Systems and Economics Unit, Health Systems and Infectious Diseases Division, ICDDR, B: Center for Health and Population Research, GPO Box 128, Dhaka-1000, Bangladesh; 3 Nossal Institute for Global Health, The University of Melbourne, Carlton, Victoria 3010, Australia

## Abstract

**Background:**

Little is known about the cost recovery of primary health care facilities in Bangladesh. This study estimated the cost recovery of a primary health care facility run by Building Resources Across Community (BRAC), a large NGO in Bangladesh, for the period of July 2004 - June 2005. This health facility is one of the seven upgraded BRAC facilities providing emergency obstetric care and is typical of the government and private primary health care facilities in Bangladesh. Given the current maternal and child mortality in Bangladesh and the challenges to addressing health-related Millennium Development Goal (MDG) targets the financial sustainability of such facilities is crucial.

**Methods:**

The study was designed as a case study covering a single facility. The methodology was based on the 'ingredient approach' using the allocation techniques by inpatient and outpatient services. Cost recovery of the facility was estimated from the provider's perspective. The value of capital items was annualized using 5% discount rate and its market price of 2004 (replacement value). Sensitivity analysis was done using 3% discount rate.

**Results:**

The cost recovery ratio of the BRAC primary care facility was 59%, and if excluding all capital costs, it increased to 72%. Of the total costs, 32% was for personnel while drugs absorbed 18%. Capital items were17% of total costs while operational cost absorbed 12%. Three-quarters of the total cost was variable costs. Inpatient services contributed 74% of total revenue in exchange of 10% of total utilization. An average cost per patient was US$ 10 while it was US$ 67 for inpatient and US$ 4 for outpatient.

**Conclusion:**

The cost recovery of this NGO primary care facility is important for increasing its financial sustainability and decreasing donor dependency, and achieving universal health coverage in a developing country setting. However, for improving the cost recovery of the health facility, it needs to increase utilization, efficient planning, resource allocation and their optimum use. It also requires controlling variable costs and preventing any wastage of resources.

## Background

The Constitution of Bangladesh made an obligation for the Government of Bangladesh to ensure good health for all its citizens. However, its average expenditure on health is little more than 1% of its gross domestic product [1]. As a signatory of the Alma Ata Declaration (1978) Bangladesh adopted primary health care approach to deliver universal health coverage to its people. The global community observes greater commitment and resources for global health over the last 30 years after Alma Ata; however, the global commitment did not necessarily result in sustainable health improvements for the poor [2]. In the low and middle income countries, there is a renewed interest in primary health care because of inequalities in health, inadequate progress towards the Millennium Development Goal (MDG) targets, shortage of human resources, and weak and fragmented health systems [3]. Without strengthening of primary health care services significantly, the health-related MDGs will not be achieved in the most low-income countries by 2015 [4]. In Bangladesh, given the current maternal mortality ratio of 322 per 100,000 [5] and under-5 mortality rate of 65 per 1000 [6], the role of primary health care service delivery is critical to achieving the health-related MDG goals by 2015 and beyond. Analysis of 30 low-income countries showed that over the period of 1990-2006, Bangladesh was able to achieve on average 4.8% yearly reduction in under-5 child mortality with a position 16^th ^compared to the first position of Thailand with 8.5% average yearly reduction in the same indicator [7]. Case studies on Pakistan and Uganda showed that primary health care could make a significant difference to maternal, newborn and child health (MNCH) and mortality outcomes. So, based on the observed evidence, countries should prioritize primary health care to strengthen MNCH services in order to reach MDG targets reducing maternal and child mortality [8] since MNCH is at the center of primary health care [9].

With a population of 150 million, Bangladesh is the 7^th ^most populous country in the world. Sixty percent of health care services occur in the private sector [10], however, an expansive network of primary, secondary and tertiary government health facilities exists. As of March 2010, 2506 non-government organizations (NGOs) [11] are present in Bangladesh and out of them 48% big and 60% small NGOs [12] are providing health care services in the rural, urban and semi-urban areas where government's services are inadequate. NGOs and private providers are doing better than the public sector providers both in the delivery of maternal and child health services (antenatal care: 53% vs. 44%) and institutional delivery (8% vs. 7%) [6]. In fact, the NGO health facilities are substantiating government's program throughout the country. Among the NGOs in health sector, the Building Resources Across Community (BRAC) is a reputed provider in community-based direct service delivery. Just nine months after the inception of BRAC in the post-liberation war of Bangladesh, it initiated its health interventions through health care facilities. Along with other development interventions like education and microfinance programs BRAC always had preventive, promotional, curative and rehabilitative grassroots health programs in its community-based development programs. Reflecting on past experiences, BRAC restructured its health interventions in order to cope with the demands of national priorities and policies. Based on the experiences of past success, BRAC health program has evolved and responded to emerging national health problems. As a continuous commitment of investing in human capital development, BRAC opened static health facilities called *Shushastha *(good health) in 1995 in order to serve as a back up to BRAC's existing community-based MNCH, tuberculosis and other health interventions through providing curative health services.

The *Shushastha *is based on the philosophy of primary health care approach though it offers curative health services [13]. The justification behind such an intervention originated from growing community demand for quality clinical services at minimum costs. BRAC community health workers found that community members did not have access to affordable and good quality medical services leaving them with little or no options for seeking health care services [14]. The objective of establishing the BRAC *Shushastha *was to develop a financially and programmatically sustainable model that provides clinical services for complicated and referral cases identified in the community. It provides antenatal care, simple delivery with obstetric care for emergency cases, postnatal care, family planning both clinical and non-clinical and other reproductive health services, including treatment of reproductive tract infections through both outpatient and inpatient services. It also provides pathology laboratory services and medicines. In 1995, the *Shushastha *system was supported by a five-year grant and in 2000, BRAC developed a financial sustainability focused strategy through cost recovery. Later, BRAC was compelled to close some of the *Shushasthas *due to lack of sustainability and hence cost recovery is an issue which is yet to be achieved.

In the discourse of financial sustainability and reducing donor dependency on critical service provisions and appropriate set service prices cost recovery is a major concern for any health facility. Despite the availability of general revenue for spending in health care the governments of developing countries are increasingly focusing on the cost recovery of the health facilities in order to mobilize more resources, improve equity and increase the efficiency of health facilities which, in fact, generated a huge debate over health financing policy and the cost effective primary health care in developing countries [15]. It is a common view of policy makers in the health sector that cost recovery is a necessary component in improving the quality and financial sustainability of health services [16]. Experience from Mauritania in early nineties also showed that cost recovery led to an increase in the amount of financial resources available in health facilities; contributed in the improvement of the quality of health care and the efficiency of the health systems provided fair supply of essential drugs and motivated staff [17]. Evaluation of Niger's experience in the Integrated Management of Childhood Illness also showed that a cost recovery system succeeded in increasing the availability of essential drugs [18]. The targeted BRAC health facility in this study is a primary health care facility and cost recovery is of much concern for its sustainability in providing quality, efficient and equitable services for the poor and the under privileged communities. These are important grounds to care about whether the facility does recover its costs sufficiently or not.

There are currently 48 *Shushasthas*, of which seven are upgraded facilities providing emergency obstetric care (EmOC), in 18 rural districts of Bangladesh. In the course of years it was observed that some complicated pregnancies could not be managed at the *Shushasthas*. Therefore, an upgraded *Shushastha *was planned in each district to offer EmOC and interlinked with a network of other *Shushasthas*. The study focused on cost recovery of an upgraded *Shushastha*, located at Gazipur, the district town next to the capital city, Dhaka. In fact, it is a 17-bed mini maternal and child health hospital, which provides both inpatient and outpatient services, including medicine and pathology services. Among the inpatient services, it mainly provides EmOC and other minor surgeries excluding ear, nose and throat, eye and orthopedics. For outpatients, the health facility mainly provides consultation, medicine and pathological services. It works as a community referral center and the BRAC community health workers, known as *Shasthya Shebikas*, usually refer the patients to this facility from the community. During the period of July 2004 - June 2005, this upgraded health facility employed three doctors, seven family welfare visitors (FWVs), two laboratory technicians (LTs), five traditional birth attendants (TBAs), a ward boy (WB), two cooks, a night guard and an accountant. Their average working hours per day is 12 hours. For caesarean surgery, there is no permanent anesthetists and they are usually hired from outside, on call. It has no nurses and the FWVs provide the services of nurses. This BRAC health facility has its own classifications of charges for the patients in terms of their socioeconomic conditions. At community level, BRAC program has village organizations (VOs) and the health facility charges VO members half the cost than that of non-VO members. For example, for outpatient consultations, VO members pay only Bangladeshi Taka (BDT.) 25 (= US$ 0.43) whereas the Non-VO members have to pay BDT. 50 (= US$ 0.86).

Earlier, several studies [19-21] were conducted at the different level of public health and NGO facilities in Bangladesh in order to estimate the outpatient and inpatient services. One of the main limitations was that these studies estimated only recurrent costs and underestimated the unit costs of the health care services. Also it did not include drug costs considering the complicacy of its estimation [21]. In the last several years, a significant number of costing studies were conducted in developing and developed countries as well and most of the studies highlighted issues of efficiency [22-26]. This study estimated the cost recovery of a BRAC upgraded health facility, outlining its outpatient and inpatient services and, therefore, tried to explain its financial sustainability. This costing exercise has significant methodological implications for estimating the cost recovery ratio of primary health care facilities in the government, private and NGO sectors which, will assist them to become truly financially sustainable by reducing donor dependency, rendering the better quality and equitable services for the people.

## Methods

### Selection of facility

The study was designed as a case study covering a single NGO health facility. The health facility was purposively selected from seven BRAC upgraded facilities providing comprehensive EmOC. This particular upgraded health facility at Gazipur district was chosen because of convenience given the limited study period and budget. In fact, BRAC's upgraded *Shushasthas *are typical and a close representation of government and private primary health care facilities, providing similar services although the volume of patients varies.

### Methodology of costing

The costing exercise was conducted to obtain the costs of running the health facility from the provider's perspective. Therefore, the costs estimated for the provision of inpatient and outpatient services did not include the costs incurred by patients when obtaining care. All relevant resources used for the delivery of inpatient and outpatient services were accounted for following the 'ingredients approach' [27-29]. The 'ingredient approach' is a standard costing methodology where the researchers observe the delivery of health services and list all the resources or inputs used in the service delivery process. The method quantifies all the inputs used in the service delivery process, irrespective of who provided the input or how the inputs were paid.

### Collection of cost information

A structured questionnaire was administered at the selected BRAC health facility by a survey team to collect information on all the resources and inputs. Data collection involved interview of facility staff, extraction from facility records, and observation on the use of space, equipment, machinery, medical supplies and furniture for inpatient and outpatient services. Staff members of the health facility were interviewed to assess the percentage of total time involved in inpatient and outpatient services, and types of activities performed for which there were no reliable records. Costs were not collected by sub-service categories; rather, all costs were simply stratified by inpatient department (IPD) and outpatient department (OPD), with the aim of the research to estimate the cost recovery of the facility. However, costs of some major services were collected. Data collection was conducted between November 2005 and January 2006, covering the reference period of July 2004 - June 2005. The types of information on resources/inputs collected with the help of the structured questionnaire were classified as fixed and variable costs.

#### Personnel

The questionnaire included all the personnel who worked at the facility during the reference year. Personnel costs were mainly collected from the information available on the salary register. Here, the medical doctors, FWVs and TBAs who directly provide health services were included in the category of service providers. Other staff members were considered as non-service providers. This group is mainly engaged in supporting the service providers and in the administration and maintenance activities of the facility. Information on the total amount of salary and benefits paid to the staff during the reference year was collected.

#### Building

Information on the area of the facility was collected. A diagram of the facility floor plan was included in the pages of questionnaire. In the diagram, the functions normally carried out were recorded for each of the rooms (e.g. outpatient consultations, pharmacy, pathological laboratory, residence of the staff, inpatient service, store rooms, patient waiting room and operation theater).

#### Furniture

Information on all types of furniture in use of the health facility was collected based on reported percentage of time used for inpatient and outpatient services. Their numbers, life expectancy and current market price were collected.

#### Machineries and equipments

Information on all types of equipment and instruments in use for IPD and OPD services with their number, life expectancy and current market price were collected.

#### Supplies

Information on all types of supplies including drugs, non-drug medical items, non-drug non-medical items by IPD and OPD were collected.

#### Operational cost

Information on all operational costs including transportation, utilities, and maintenance of the facility were collected. Referral bills from nurses and anesthetist bills were also included in the operational costs.

#### Pathological laboratory

Information on the capital items used in pathology including their number, current market price and life expectancy were collected. Information on recurrent items including their amount and price during the reference year was extracted from the stock register. For both capital and recurrent items, the LTs were interviewed to figure out the percentage of time used for inpatient and outpatient services.

#### Income and utilization

Information on total income and utilization (total number of inpatients and outpatients) of the reference year was derived from the registers and checked against the monthly financial statements, in consultation with the doctor in charge of the facility and the accountant. In practice, the facility records daily income, utilization and expenditures in its register and prepares a monthly financial statement at the end of the month to submit to the BRAC Head Office.

### Valuing the inputs and sensitivity analysis

Depreciation of capital assets was estimated in order to calculate annual capital costs. For this, the market price of 2004 (replacement value) was calculated and the value was annualized using the 5% discount rate [28]. A sensitivity analysis of the estimates was also performed using the discount rate of 3% [30] to test the robustness of the cost estimates.

### Allocation of costs for inpatient and outpatient services

Personnel cost was allocated based on the percentage of time spent by the providers and non-providers for inpatient and outpatient services. The cost of capital items (e. g. machinery, equipment and furniture) was allocated to inpatient and outpatient services, according to time of their use and location of services. Facility space cost was apportioned in proportion to the area used for inpatient and outpatient services. Table 1 details the allocation procedure. Operational costs, including utilities and the maintenance of the facility were distributed among inpatient and outpatient services according to the proportion of users. Pharmacy costs were apportioned according to the proportion of total drug cost for inpatient and outpatient services. After completing all the tests and examinations in the outdoor, the patients are admitted and the health facility considers the pathology laboratory costs for the OPD, in practice. The kitchen is mainly used for inpatient services, so, all costs related to kitchen services were allocated to inpatient services.

**Table 1:** Allocation of costs for inpatient and outpatient services, BRAC *Shushastha*, July 2004-June 2005

Cost category	Allocation procedure
Personnel	Proportion of time spent for inpatient and outpatient services
Equipment & machinery	Use of equipments and machineries by inpatient and outpatient services
Furniture	Use of furniture by inpatient and outpatient services
Space Rent	Proportion of floor space used by inpatient and outpatient services
Supplies	Proportion of supplies used by inpatient and outpatient services
Operational costs	Proportion of utilities and maintenance by inpatient and outpatient services
Kitchen	100% to inpatient services
Transports	Transport costs incurred for inpatient and outpatient services
Laboratory	100% to outpatient services
Pharmacy	Proportion of total drug costs used by inpatient and outpatient services

### Estimation cost per patient and cost recovery

Microsoft Excel was used to facilitate data processing and analysis. Costs were expressed in local currency (Bangladeshi Taka) during data collection and converted into US$ in the article. A mid-point exchange rate (31^st ^December 2004, 1BDT. = US$ 0.01718) of the reference year was used for currency conversion, following http://www.oanda.com. The total number of inpatients and outpatients during the reference year were the outputs. Total costs were obtained for inpatients and outpatients, separately adding all the costs (capital, recurrent, building rent, personnel, supervision, training, and supplies), and unit costs for inpatient, outpatient and cost per patient were estimated. Finally, cost recovery ratios were calculated dividing the total income by the total costs for inpatient, outpatient and for the combined estimate as well.

## Results

### Personnel costs

Three medical doctors, seven FWVs and five TBAs were working as the service providers out of 22 staff of the facility during the reference year. The direct service providers absorbed 77% of total personnel costs and the rest of the personnel costs were incurred for non-service providers (LTs, WB, cook, night guard and accountant). In both IPD and OPD, the personnel cost of the service providers was three times higher than personnel cost of the non-service providers (Table 2). Mean yearly salary for the service providers was US$ 937 while the same for the non-service providers was US$ 591 during the reference year.

**Table 2:** Distribution of personnel costs, BRAC *Shushastha*, July 2004-June 2005

Category	Provider	IPD% (US$)	OPD% (US$)	Total% (US$)
Service provider	15	81.44 (5067.11)	75.10 (8984.07)	77.27 (14051.18)
Non-service provider	7	18.56 (1154.48)	24.90 (2979.29)	22.73 (4133.77)
Total	22	100 (6221.59)	100 (11963.36)	100 (18184.95)

### Capital costs

Equipment and machinery constituted the major share (52%) of the capital costs of the facility and it was the highest (78%) when only inpatient service costs (Table 3) were considered. The share of pathology laboratory was 19% and for inpatient services, it was zero. Furniture items constituted 18% of the total capital costs. The health facility has only one vehicle (motor bike) and the medical officer uses it. Examples of some maternal health IPD capital items were anesthesia machine (cost: US$ 653, life expectancy: 5 years); operation theater light (cost: US$ 515, life expectancy: 3 years); operation theater bed (cost: US$ 928, life expectancy: 5 years); oxygen cylinder (cost: US$ 137, life expectancy: 5 years); sucker machine (cost: US$ 430, life expectancy: 4 years); autoclave machine (cost: US$ 206, life expectancy: 5 years); refrigerator (cost: US$ 378, life expectancy: 5 years); patient bed (cost: US$ 584, life expectancy: 5 years); and dilation & curettage set (cost: US$ 77, life expectancy: 2 years).

**Table 3:** Distribution of capital costs, BRAC *Shushastha*, July 2004-June 2005

Assets	IPD% (US$)	OPD% (US$)	Total% (US$)
Furniture	13.81 (880.29)	26.40 (901.92)	18.21 (1782.22)
Equipment & machinery	77.81 (4959.40)	4.71 (161.05)	52.30 (5120.45)
Pathology lab items	0.00 (0)	54.93 (1876.42)	19.17 (1876.42)
Vehicles	1.40 (89.29)	2.61 (89.29)	1.82 (178.59)
Others	6.97 (444.41)	11.34 (387.52)	8.50 (831.93)
Total (annualized)	100 (6373.40)	100 (3416.21)	100 (9789.61)

### Recurrent costs

Drugs accounted for the largest share (42%) of the recurrent costs of the health facility, followed by the operational costs (27%) (Table 4). The operational costs included maintenance, utilities, stationeries, entertainment cost, nurses' referral bill and anesthetists' bill. The anesthetist's bill was alone 55% of the total operational cost of the facility. In inpatient services, drug costs were highest (44%) followed by the operational cost (30%). Similarly in outpatient services, drugs accounted for the highest cost (34%) followed by the pathology cost (26%). The recurrent costs of the laboratory constituted 45% of the total costs of pathology. Almost all the non-drug medical items (needle, savlon, cannula, catgut, etc.) were used for inpatients and therefore these were not estimated for outpatient services.

**Table 4:** Distribution of recurrent costs, BRAC *Shushastha*, July 2004-June 2005

Category	IPD% (US$)	OPD% (US$)	Total% (US$)
Drug	44.15 (8504.99)	34 (2048.56)	41.73 (10553.54)
Non-drug medical	22.52 (4338.72)	0 (0)	17.16 (4338.72)
Non-drug non-medical	3.55 (683.14)	20.37 (1227.63)	7.56 (1910.77)
Operational costs	29.78 (5737.56)	19.22 (1158.15)	27.27 (6895.71)
Pathology (recurrent)	0 (0)	26.41 (1591.11)	6.29 (1591.11)
Total	100 (19264.40)	100 (6025.45)	100 (25289.85)

The health facility is in a rented building and the rent per square foot was BDT. 39.27 (US$ 0.67). The total rent of the building for the reference year was US$ 3917. The ground floor was used mainly for doctors' outpatient consultation, pharmacy and the pathology laboratory. The first floor was used as inpatient cabin, interdepartmental unit and labor room and the third floor for operation theater (OT), kitchen and residence of FWVs. More than three-quarters (79%) of the building was occupied for inpatient services.

### Fixed vs. variable costs

The variable costs comprised more than three-quarters of the overall costs of the health facility (Table 5). Annualized costs of all capital items (furniture, machinery and equipment) constituted the highest part (17%) of the fixed items. Among the variable items, personnel accounted for the highest costs (32%) next to the drug costs (18%). In the IPD, the proportion of drug costs was highest (24%) and in the OPD, personnel constituted the highest (53%) costs. The total variable cost (73%) was more than two times higher than that of the fixed cost (27%) within the IPD. Similarly, total variable cost (80%) was four times higher than that of fixed costs (20%) for outpatient services.

**Table 5:** Distribution of fixed and variable costs, BRAC *Shushastha*, July 2004-June 2005

Category	IPD% (US$)	OPD% (US$)	Total% (US$)
*Fixed costs*			
Space rent	8.81(3099.91)	3.63 (817.13)	6.81
Capital items (annualized)	18.12 (6373.40)	15.27 (3416.21)	17.01
Supervision	0.38 (133.66)	0.60 (133.66)	0.46
*Total fixed costs*	*27.31 (9606.97)*	*19.52 (4367.00)*	*24.28 (13973.97)*
*Variable costs*			
Personnel	17.69 (6221.59)	53.47 (11963.36)	31.60
Training	0.23 (79.74)	0.09 (19.94)	0.17
Drug	24.18 (8504.99)	9.16 (2048.56)	18.34
Non-drug medical	12.34 (4338.72)	0.00 (0)	7.54
Non-drug non-medical	1.94 (683.14)	5.49 (1227.63)	3.32
Operational costs	16.31 (5737.56)	5.18 (1158.15)	11.98
Pathological (recurrent)	0.00 (0)	7.11 (1591.11)	2.76
*Total variable costs*	*72.69 (25565.73)*	*80.48 (18008.75)*	*75.72 (43574.48)*
Total costs	100 (35172.70)	100 (22375.74)	100 (57548.44)

**Figure 1:**
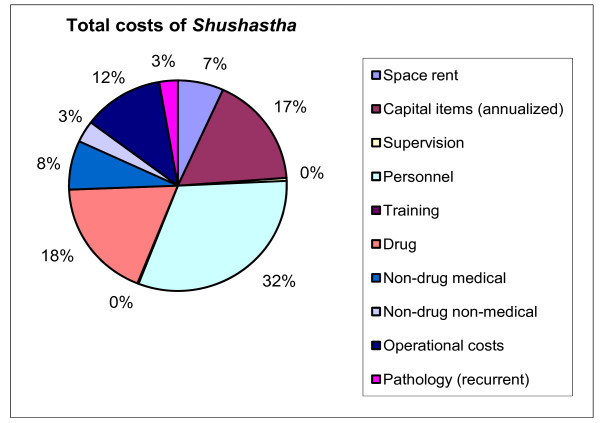
**Total costs of BRAC *Shushastha*, July 2004-June 2005**.

Total cost distribution shows (Figure 1) that 32% of total cost was for personnel during the reference year. Next, drugs accounted for 18% of the total costs, followed by the capital costs (17%). Operational costs alone constituted 12%. The other categories of costs varied from 0%-8%.

### Total utilization, revenue and costs per patient

**Figure 2:**
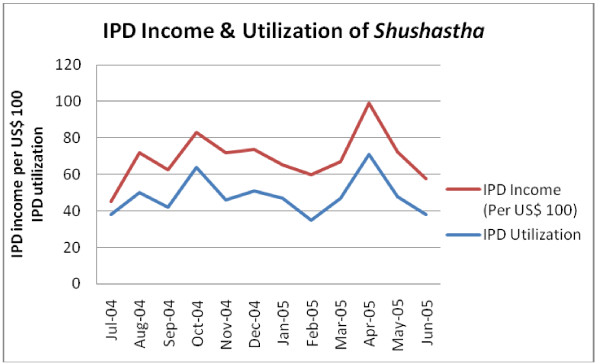
**IPD income and utilization of BRAC *Shushastha*, July 2004-June 2005**.

**Figure 3:**
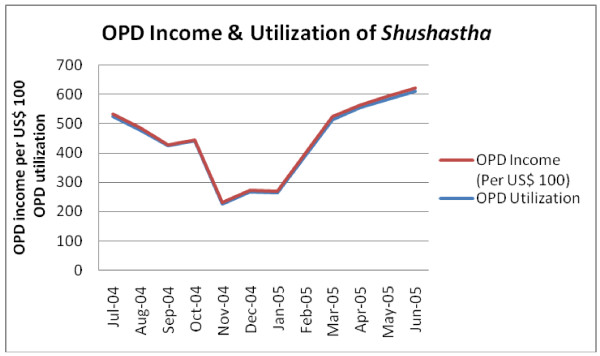
**OPD income and utilization of BRAC *Shushastha*, July 2004-June 2005**.

A total of 5857 patients visited the health facility during the reference period and 90% of them were outpatients (Table 6). Usually, patients with complicated problems came for inpatient services. Common inpatient services available at the facility were cesarean section (cost: US$ 108); dilation & curettage (D&C) (cost: US$ 43); menstrual regulation (MR) (US$ 14); and normal delivery (US$ 14). The numbers of outpatients were 90% but they only contributed 26% of total income of the facility. Over the reference period of 12 months, the health facility showed seasonal variations over both IPD and OPD utilization and income (Figure 2 & Figure 3). In April 2005, the IPD of the health facility treated the maximum number of patients and earned the highest amount of income. The OPD utilization and income showed U-shape trend over the period. In the months of May and June 2005, the OPD served the highest number of patients and earned the highest as well. The cost per inpatient was US$ 66.96, which was about 16 times of cost per outpatient. However, irrespective of inpatient and outpatient costs, the average cost per patient of the facility was US$ 9.83. If capital costs are excluded from the total costs, the average cost per patient would be US$ 8.15. Based on a sensitivity analysis using a 3% discount rate for the capital items, the average cost per patient was US$ 9.75. The sensitivity analysis revealed that the average cost per patient was not significantly sensitive to changes in the discount rate.

**Table 6:** Distribution of utilization and income, BRAC *Shushastha*, July 2004-June 2005

Category	Utilization% (Patient)	Income% (US$)
IPD	9.85 (577)	73.71 (25215.07)
OPD	90.15 (5280)	26.29 (8995.69)
Total	100 (5857)	100 (34210.76)

### Cost recovery ratio

The cost recovery ratio for IPD was 72% while it was 40% for OPD for the reference year (Table 7). The average cost recovery of the health facility for the reference year was 59%. Excluding the capital costs, the average cost recovery ratio of the health facility for IPD would be 88%, while it would be 47% for OPD. For the same, the average cost recovery ratio of the health facility would be 72%. The sensitivity analysis revealed that the cost recovery of the health facility was not sensitive to changes in the discount rate (3%) as it was less than 1% higher than the estimate calculated in the main analysis.

**Table 7:** Estimation of cost recovery ratio, BRAC *Shushastha*, July 2004-June 2005

Category	IPD (US$)	OPD (US$)	Total (US$)
Income	25215.07	8995.69	34210.76
Costs	35172.70	22375.74	57548.44
*Cost recovery ratio*	*71.69*	*40.20*	*59.45*

Cost recovery ratio excluding capital costs			

Costs	28799.30	18959.53	47758.83
*Cost recovery ratio*	*87.55*	*47.45*	*71.63*

## Discussion

The cost recovery of a primary health care facility is of great concern for its financial sustainability and for providing quality, efficient and equitable health services to the community. In terms of cost recovery, IPD services contributed more than OPD services. Nearly, three-quarters of the total revenue came from the IPD services of the facility. The cesarean section is the most important revenue generating service among the IPD services while other services like normal delivery, D&C, MR, and outpatient consultations also played a significant role in increasing the overall cost recovery of the facility.

Increasing the efficiency of the health facility through controlling existing costs and optimizing the use of available resources is important for improved cost recovery. Among the existing cost categories, personnel costs were the highest, which was about one-third of the total costs of the facility. Although personnel cost was a large component for IPD and OPD services, the link between staff productivity and unit cost was not explored. The study also did not address the issue of efficiency. In fact, analysis was not done to figure out whether the numbers of health care providers at the facility were appropriate for the service volume of the facility. Time motion data was not available to find the amount that health care providers spent on unoccupied or personal activities. The effective and efficient utilization of personnel would positively affect the utilization of the health facility. It was also found that efficient use of unutilized time of the providers would help to reduce the costs of providing services [31]. However, it can be recommended for the health facility that more efficient and effective personnel management may reduce the operational cost of the health facility.

Drugs were the second major component of all costs categories. Drug companies directly supplied the required drugs to the BRAC facility through their sales representatives. Compared to the government drug procurement system for primary health care facilities like sub-district level Upazila Health Complex (UHC) and Union Family Welfare Center, BRAC's health facilities enjoyed much more direct, functional, transparent and a quicker drug supply system. The government system always involves lengthy and complicated procedures for drug procurement. In the BRAC health facility, clear records of drug usage in the registry were maintained separately for IPD and OPD services, and there were no anomalies found in the drug registers examined. However, given the higher percentage of drug costs compared to other costs, it was anticipated that misuse of drugs may be the reason for higher drug costs. Further studies are needed to explore the underlying reasons. A lower pathology cost of the facility also supports this assumption. In order to minimize the gap in cost recovery, there is a need for efficient use of all supplies and utilities, including drugs, to prevent wastages.

In other words, given the high magnitude of variable costs, there might be scope to control variable costs and to maximize the cost recovery of the health facility. Specifically, the allocation of resources (personnel, supplies and operational costs) could be reviewed to reduce the variable costs of the facility.

This study offers an opportunity to compare the unit costs of BRAC health services to available service costs of the same kind. The unit cost of normal delivery at Rural Service Delivery Partnership (RSDP) supported NGO facilities was US$ 2.37 [32] while it was US$ 5.72 [33] at the Urban Family Health Partnership (UFHP) supported NGO facilities. The unit cost of normal delivery was US$ 2.17 - US$ 4.70 at the government primary health care facilities and US$ 9.04-10.13 in other NGO facilities [29]. The unit cost of normal delivery at the BRAC facility was US$ 14 which seems quite higher than other available estimates. The unit cost of c-section at the government sub-district level UHC was US$ 6.71 while in other NGO facilities it was US$ 79.59 [29]. The unit cost of c-section at the BRAC health facility was US$ 108. Although drugs and supplies were provided free of cost at the government facilities, BRAC's c-section cost was still higher. Annual average cost per patient of US$ 9.83 in our study was much higher than the projected cost per patient for maternal health of US$ 3.6 as part of the essential service package (ESP) of the public sector of Bangladesh [34].

The constraint of using this study is the generalizabilty of its findings because it was conducted at a single facility and heath facilities may not have homogeneous cost components. It is too small to generalize its findings to the country as a whole although this BRAC health facility is a close representation of the government and other NGO primary health care facilities. The findings of this study need to be verified in a larger costing study. There is always a certain level of approximation and arbitrariness in various allocation keys in this costing methodology - an intrinsic limitation. The differences found between the cost recovery ratios estimated by this study and BRAC might be due to variation in methodology or may be for the specific reference year. However, the reasons for such variation need to be explored through further studies. Stratifying costs only by IPD and OPD and not by subservice categories creates a missed opportunity to consider which major sub-service categories incur the greatest cost and which could also guide understanding of price setting and profit margins. However, this study should be considered a significant initiative for measuring financial sustainability of primary health care facilities in Bangladesh and other developing country contexts.

## Conclusion

This study gives us with the insights into the financial sustainability of an NGO primary health care facility in a developing country setting, through examination of costs, revenue and cost recovery status. The information on factors that contributed to variation of costs per patient or variation in terms of contribution of IPD and OPD services in cost recovery are critical to the operations of such health facilities. The gap found between costs and the cost recovery of the health facility needs to be explained carefully. At the same time, pragmatic mechanisms need to be developed to minimize this gap for sustainable health services. More specifically, a strategy should be developed to improve the efficiency of the facility. Program managers may look into ways of increasing facility utilization, maximizing use of staff and encouraging more rational use of drugs. Special emphasis should be placed on increasing utilization of IPD services by arranging additional IPD services.

This study can contribute to formulating macro level policy and health sector reform strategies in light of cost analysis and cost recovery of primary health care facilities. The findings can have implications for program budgeting, reducing donor dependency and also for the improvement of services. Planners use cost data in designing new health care interventions and this study may contribute to designing similar kinds health facilities for BRAC in other parts of the country. In the light of current maternal and child mortality scenario of Bangladesh, and addressing the challenges to meet health-related MDG targets by 2015, financially sustainable primary health care facilities are critical and this current study is certainly an important initiative in an under-researched area by providing estimates of cost recovery of an NGO primary health care facility. Despite the constraints in making the findings generalizable, the study has significant methodological implications in estimating the cost recovery ratios for the primary health facilities both in government and private sector including NGOs. Finally, it offers basis for undertaking cost-effectiveness or cost-benefit analysis for similar primary health care facilities.

## Competing interests

The authors declare that they have no competing interests.

## Authors' contributions

KA designed the study, analyzed the data, and wrote the manuscript. SA guided KA throughout the design, analysis and preparing and editing the manuscript. Both authors have read and approved the final manuscript.
